# Spatio-temporal evolution of port opening in China's 40 years of reform and opening-up period

**DOI:** 10.1371/journal.pone.0220912

**Published:** 2019-08-12

**Authors:** Xiaoshu Cao, Shengchao Li

**Affiliations:** School of Geography and Planning, Sun Yat-Sen University, Guangzhou, Guangdong, China; University of Shanghai for Science and Technology, CHINA

## Abstract

In the past 40 years of reform and opening-up, China has developed from an economically closed country to a country that is highly dependent on foreign trade. From the perspective of spatiotemporal evolution, we analyze how port opening promoted China's reform and opening-up process. First, the port development process is divided into four periods. In the start-up period, the pilot open port policy created a platform for foreign cooperation and exchange. During the expansion period, port openings promoted the continuous optimization of the trade structure. In the cooperation period, port openings corresponded with the adjustment of China's overall industrial structure. During the optimization period, port openings provided guarantees for the implementation of a national development strategy. Second, we analyze the distribution of ports and their relationship with cross-border logistics and passenger flow. Based on data of foreign trade and passenger flow, a port openness degree measurement model includes port logistics intensity, passenger flow intensity and port city foreign-trade volume is constructed. There are significant types, geographical differences and grade differences of ports' openness.

## Introduction

The definition of port of entry by the General Administration of Customs of China is ports, airports, stations, cross-border passages, etc., for people, commodities, goods and vehicles directly crossing the national border or the customs boundary. There are five types of ports: seaports, riverports, airports, roadports and railports. They are divided into first class ports of entry and second class ports of entry [1]. In the past 40 years of reform and opening-up, China has developed from an economically closed country to a country that is highly dependent on foreign trade. The dependence on foreign trade has increased from 9.65% in 1978 to 33.88% in 2018. In 2006, it reached as high as 64.24%. As national gateways, ports of entry are increasingly important for China's opening and connecting functions [2–4]. Research on port of entry has focused on international trade and economic development, international transportation and supply chains [5–8], and port characteristics [9–13]. Research on China’s ports of entry mainly focuses on the geographical environment and development level of the border ports system [14–20], the port spatial structure in different regions [21–24], the port development model [16, 25–28], the function of specific ports and their regional cooperation [29–32], the relationship between the ports and the hinterland [14, 33–37], and the historical development of a port's development process and its impact on the regional economy [38–40]. In general, the studies on China’s port system are mainly macro descriptions of the current state. There are few studies on the spatiotemporal evolution of ports of entry. Research subjects are mainly coastal ports, and there are few comprehensive studies on multiport types. Therefore, it is necessary to comprehensively analyze the relationship between various types of ports and the country's opening to the outside world from the perspective of spatiotemporal evolution. This paper first analyzes the interaction between the port development process and China's opening up policy from a time perspective, and then analyzes port distribution and its relationship with cross-border logistics and passenger flow from a spatial perspective.

The degree of openness refers to the degree to which a country or region's economy is open to the outside world. It is a multilevel comprehensive index covering foreign trade, foreign investment, foreign economic cooperation and exchanges. The study of openness degree originated from measuring foreign trade dependence. Early studies mostly used foreign trade dependence as a measure of openness [41]. In many studies, the narrow sense of openness degree is equivalent to the foreign trade dependence. However, the broad sense of openness degree refers not only to foreign trade dependence but also to the dimensions of financial openness, investment openness, production openness, technology openness, and openness to immigrants. Based on national restrictions on imports and exports, the World Bank divided countries into four levels according to their openness degree [42]. Sachs and Warner established the SW (Sachs & Warner) indicator system, which includes comprehensive tariff rates, nontariff barriers, socioeconomic patterns, monopoly, and smuggling factors to measure the degree of national economic openness [43]. Lemer first estimated bilateral trade intensity and then used the average of the difference between the predicted and actual values as the trade openness indicator [41]. Stewart used the gravity model as a basis to predict trade flows, and he used the difference between actual and predicted trade flows as an indicator of trade openness [44]. Based on international trade theory, Dollar used the exchange-rate distortion index to reflect the openness to foreign trade [45]. Studies on openness have been continuously improved, and it is still a widely-used method to reflect the degree of openness through the dependence on foreign trade. This method is used because the data on foreign trade and GDP are more accessible and have better comparability among different regions. Therefore, the measure of port openness should also follow the criteria of data availability and comparability.

The second part of this study describes the data sources and analysis methods. In the third part, based on spatiotemporal data, we analyze the development pattern of China's ports of entry and the formation process of the port open regional system in the past 40 years of reform and opening-up, and we discuss the spatiotemporal evolution of the ports of entry. The fourth part is based on the port's foreign trade logistics intensity, international passenger flow intensity and the amount of port city foreign trade—which is how the port's openness measurement model is constructed—and so we obtain the port's comprehensive openness degree. The fifth part is the conclusion.

## Data and methodology

### Data

The customs import and export data is from the enterprise import and export database of the China Customs Administration in 2015. The data includes all types of foreign-trade enterprises in mainland China, and the quantity of import and export goods. Other data comes from the 2016 China Statistical Yearbook, the China’s Ports of Entry 2016 Yearbook and the statistical yearbooks of 31 provinces (including municipalities and autonomous regions).

For the purposes of the data, in cases where a city has multiple ports, if they are the same type of ports, then they are combined into one port. This is also a common practice in government statistics. For example, Guangzhou City has three sea ports: Lianhuashan, Guangzhou and Nansha, which are combined together as the Guangzhou sea-ports; Shenzhen City has six road ports: Futian, Huanggang, Luohu, Shatoujiao, Shenzhen Bay and Wenjindu which are all combined together as the Shenzhen road-port.

### Principal component analysis

The principal component analysis method uses the idea of dimensionality reduction to perform linear transformation, and it converts multiple indicators that are originally related to each other into a few comprehensive indicators or principal components. Each principal component is irrelevant, and each principal component is a linear combination of original variables that can reflect most of the information of the original variables—the information contained therein does not overlap [46]. In this way, multiple factors that reflect the port openness degree (POD) can be attributed to several principal components, which helps simplifies complex problems.

The port openness measurement is an area that scholars have rarely studied, and there is no mature measurement standard yet. Referring to the foreign trade dependence measurement method commonly used in the study of the country's openness [47], according to the characteristics of the port's openness, we built a model suitable for port openness measurement. Ports of entry are a country's doors to the outside world. Their function is to connect internal and external markets, transport foreign trade goods, transport inbound and outbound passengers and serve the foreign trade of the hinterland cities. Therefore, the POD should be examined from the port foreign trade logistics intensity (FLI), port international passenger intensity (IPI) and port city foreign trade amount (CFA). In this paper, principal component analysis is used to calculate the POD of 207 ports through a model containing 7 indicators[48, 49] (Table 1). Through these indicators including the number of countries or regions, the volume of goods, and the number of passengers passing through, the broadness and intensity characteristics of the port opening are reflected[47–49].

**Table 1:** Variable list.

	Indicators	Unit	Minimum	Maximum	Mean	S.D.	Weight
port foreign-trade logistics intensity (FLI)	port export logistics volume (PEL)	ton	0	1.26E+08	3.42E+06	1.24E+07	1.03E-01
	port import logistics volume (PIL)	ton	0	2.87E+08	1.20E+07	3.82E+07	7.91E-02
	port external market coverage (EMC)		0	1.92E+02	2.20E+01	3.95E+01	7.08E-02
port international passenger intensity (IPI)	port outbound passengers (POP)	time	0	8.77E+07	1.12E+06	7.62E+06	2.62E-01
	port inbound passengers (PIP)	time	0	8.36E+07	1.11E+06	7.50E+06	2.62E-01
port city foreign-trade amount (CFA)	port city export trade amount (PCE)	yuan	1.38E+07	1.28E+12	1.11E+11	2.42E+11	1.25E-01
	port city import trade amount (PCI)	yuan	5.53E+04	1.67E+12	9.58E+10	2.81E+11	9.84E-02

### Spatial correlation analysis

We use local Moran's I and global Moran's I to analyze the spatial correlation of port openness. Global Moran's I is a description of the spatial characteristics of an attribute value over the entire region. The local Moran's I is used to explore whether a single region has a high or low spatial agglomeration of observations, and the contribution of each unit of the regional space to the global spatial autocorrelation. The global Moran's I is complementary to the local Moran's I. The former reflects the spatial agglomeration of the attributes, while the latter mainly analyzes the regional heterogeneity. The global Moran's I equation is as follows:

$$I=\frac{n\sum_{i=1}^{n}\sum_{j=1}^{n}w_{ij}(x_{i}-\bar{x})(x_{j}-\bar{x})}{(\sum_{i=1}^{n}\sum_{j=1}^{n}w_{ij})\sum_{i=1}^{n}{\left(x_{i}-\bar{x}\right)}^{2}}$$ (1)

Since the global Moran's I failed to reflect the regional heterogeneity characteristics, in order to further reflect the local spatial characteristics of the port's openness, the local Moran's I was introduced:

$$I_{i}=\frac{n(x_{j}-\bar{x})\sum_{i}w_{ij}(x_{j}-\bar{x})}{\sum_{i=1}^{n}{\left(x_{i}-\bar{x}\right)}^{2}}$$ (2)

In the above equations, *n* is the number of research ports; *x*_*i*_ and *x*_*j*_ are the original values of sample *i* and sample *j*, respectively; $\bar{x}$ is the sample mean; *w*_*ij*_ is the spatial weight matrix. Using the Geo DA software, the K-nearest matrix is used as the spatial weight matrix to calculate the global and local Moran's I. When *I* is positive, there is a positive spatial agglomeration, otherwise it means a negative spatial agglomeration; when *I*_*i*_ is positive or negative, respectively, the local spatial unit similarity value tends to be agglomerated or distributed, and can be visualized with LISA map[50, 51].

### Evolution tree

The cities evolution tree model was proposed by Wang and was applied to study the evolution law of Chinese cities[52]. The cities evolution tree draws on evolutionary theory in biology and expresses the multidimensional data generated by urban evolution in a simple and clear visual form. The theoretical basis for the construction of an evolutionary tree is the ergodic theorem of physics: Individual evolution constitutes group evolution, and individual evolution will follow the regularity exhibited by group evolution. The evolution tree is also a visualization method, that establishes the mapping relationship between attribute state space and a space-time pattern. In this study, the K-means clustering algorithm was used to cluster the ports according to the seven indicators required for POD measurement, and to arrange the branches and leaves so as to form a ports evolution tree. Each of the branches represents a type of port, and each leaf represents a port; these are arranged according to the POD value.

### Geodetector

Spatial stratified heterogeneity (SSH) is one of the basic characteristics of geographical distribution, and the difference of spatial distribution is often influenced by many factors. Exploring its differentiation mechanism is an important part of geography research. Geodetector is a tool for measure of spatial stratified heterogeneity and attribution of spatial patterns. The core idea is to use the difference between the sum of the variances in the classification layer and the total variance of the whole region to detect the spatial differentiation of the dependent variable and the ability of the independent variable to explain the spatial differentiation of the dependent variable[52–54]. The model is as follows:

$$q=1-\frac{\sum_{h-1}^{L}N_{h}\sigma_{h}^{2}}{N\sigma^{2}}$$ (3)

In the above equations, L is the strata of the variable Y or factor X. *N*_*h*_ and N are the number of cells in strata h (h = 1, 2, …) and the whole region, respectively. $\sigma_{h}^{2}$ and *σ*^2^ are the variances of the strata h and the Y value of the whole region, respectively. The value range of q is [0, 1]. The larger the value, the more obvious the spatial differentiation of Y. If the stratification is generated by the independent variable, the larger q value is, the stronger the explanatory power of the independent variable X on the attribute Y is, and vice versa. The geodetector q statistic can be used to measure spatial differentiation, detect interpretation factors and analyze the interactive relationship between variables. In this study, the dependent variable Y is POD, and the factor X contains 7 indicators (Table 1).

## The spatiotemporal evolution of China's ports of entry

To better understand the POD of China's ports of entry, it is necessary to analyze their spatiotemporal evolution. We divide the process into four stages according to the port opening policy and divide the ports into four port open regional systems based on geographical location.

### Four periods

**Fig 1:**
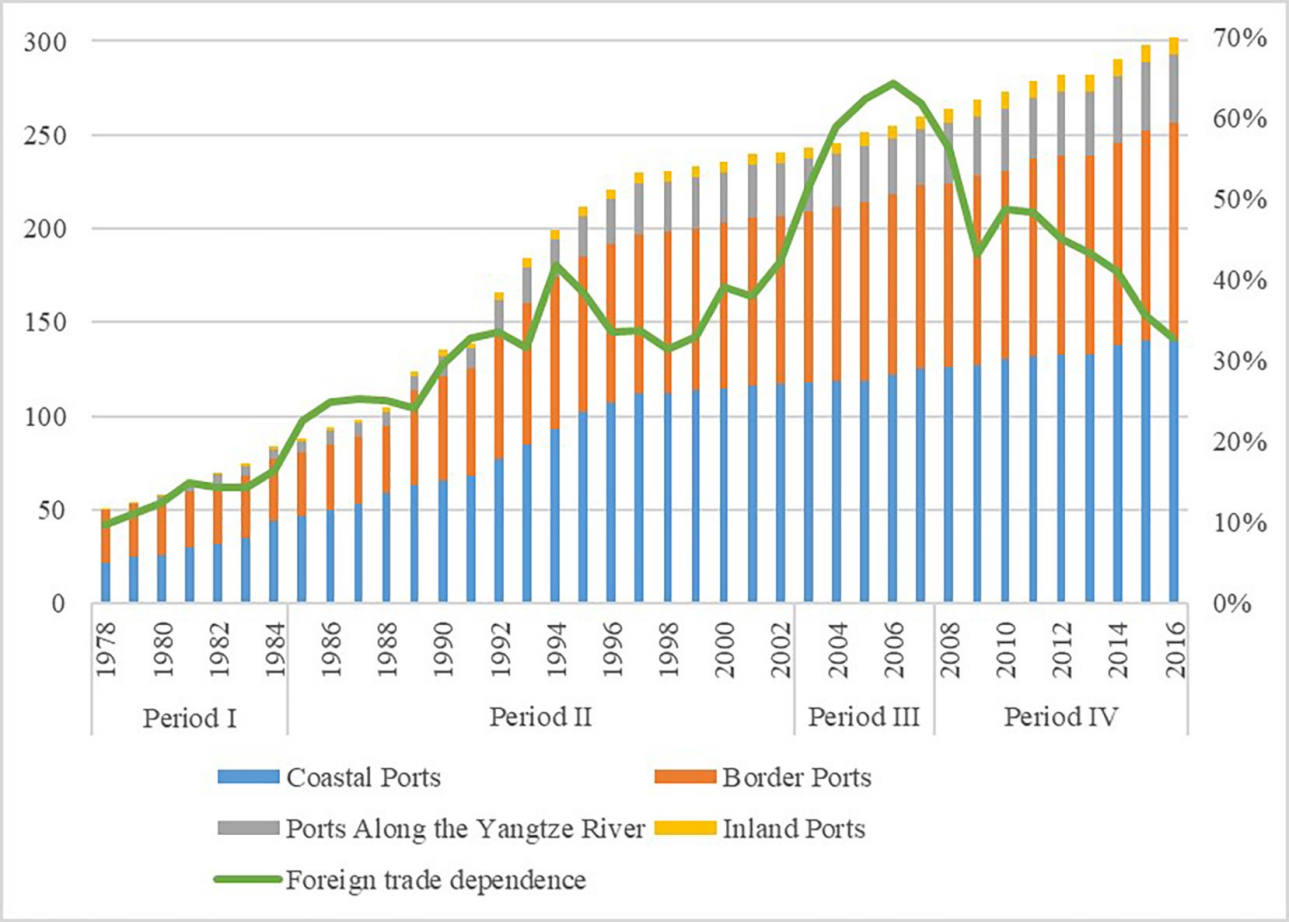
The changes in the number of ports of entry in the 40 years of reform and opening-up.

Before the reform and opening-up in 1978, China had 51 national first class ports of entry open to the outside world. Among them, water-ports (including seaports and riverports) were mainly concentrated in the eastern coastal areas and along the Heilongjiang River, railports and roadports were concentrated along the border areas, and airports were only distributed in six regional central cities. Due to the small number of ports of entry and the uneven spatial distribution, China's foreign economic ties and foreign trade development had been hindered. In the 40 years since opening-up, China's ports of entry have undergone tremendous changes. The reform and opening up policies have played a decisive role in the development of ports. In line with the policy changes, we divide the opening process into four periods (Fig 1).

The first period, from 1978 to 1984, was a startup period characterized by pilot policy programs. The Third Plenary Session of the 11th Central Committee of the Chinese Communist Party proposed the reform and opening up policies, marking the end of a long-term closure of the Chinese economy. China seized the opportunity of the industrial upgrading of the ‘four Asian tigers’ and the secondary transfer of labor-intensive industries, and began to participate in international industrial cooperation. During this period, China established special economic zones such as Shenzhen, Zhuhai, Shantou and Xiamen and opened 22 coastal ports. Relying on the comparative advantage of China’s cheap labor resources, the labor-intensive export processing industry has developed rapidly. The pilot port openings during this period provided a platform for foreign cooperation and exchanges, and supported China’s most critical and difficult period for economic restructuring. In these 7 years, China's foreign trade had an average annual growth rate of 24.11%, and 34 ports were newly opened, which brought the total to 84. The newly opened ports were mainly seaports and riverports in the eastern coastal provinces, airports in the central and eastern provinces, and relatively few roadports and railports.

The second period, from 1985 to 2002, was an expansionary period characterized by policy guidance. In 1985, the State Council promulgated the ‘Several Provisions on Port Opening’ [55], which clearly guided and expanded port opening from the national policy level. In 1992, Deng Xiaoping’s Southern Talks marked the further expansion of China’s opening up. China has seized the opportunity of the labor-intensive part of the manufacturing industry in developed countries to shift outwards and gives priority to the development of export-oriented manufacturing and high-tech industries. Taking the opening of Pudong as an opportunity, China implemented a series of policies in Shanghai to encourage opening up. The opening up of the ports also expanded inland from the coast, and the inland provinces have with conditions, gradually opened up airports and riverports. Foreign capital began to flow into the mainland on a large scale, foreign trade continued to grow, and the trade structure was continuously optimized. China’s economy rose rapidly and its overall national strength has increased substantially. These cumulative changes led China to become a member of the World Trade Organization and to participate fully in economic globalization. In these 18 years, China's foreign trade has had an average annual growth rate of 24.71%, while 157 ports were newly opened, bringing the total to 241.

The third period, from 2003 to 2007, was the period of cooperation characterized by institutional openness. With the accession to the World Trade Organization, China's opening up shifted from partial opening to institutional openness. During this period, China began to upgrade its industrial structure. The Chinese government proposed a strategy of developing a modern service industry and advanced manufacturing in the coastal areas, developing strategy of the Bohai Rim region, revitalizing of the old industrial bases in the Northeast, and developing the western region. The eastern, central and western regions were opened up according to the industrial gradient. The core goal of port openings during this period was to coordinate with China's overall industrial restructuring and ensure the implementation of its WTO commitments. This coordination not only enhanced China's comprehensive national strength but also promoted the improvement of the socialist market economic system. In these 5 years, China's foreign trade had an average annual growth rate of 26.81%, and 19 ports were opened, bringing the total to 260.

The fourth period, from 2008 to the present, is an optimization period characterized by comprehensive opening. The 17th National Congress of the Communist Party of China (NCCPC) put forward a comprehensive opening strategy of "deepening the opening up of the coastal areas, speeding up the opening up of the central areas, and optimizing the opening of the border areas. " After the 18th NCCPC, in order to adapt to economic globalization, China formulated a more proactive open strategy and paid more attention to the balance, security and efficiency of opening up. After the Third Plenary Session of the 18th NCCPC, China proposed building a new open economic system, expanding the opening of the inland borders, and promoting exchanges and complementary advantages in inland and coastal areas. Full openness is also an important condition for the implementation of national strategies, such as the Silk Road Economic Belt, the 21st Century Maritime Silk Road, and the Yangtze River Economic Belt. In the 9 years from 2008 to 2016, China's foreign trade had an average annual growth rate of 5.12%, and 42 ports were newly opened, bringing the total to 302.

### Four ports open regional systems

**Fig 2:**
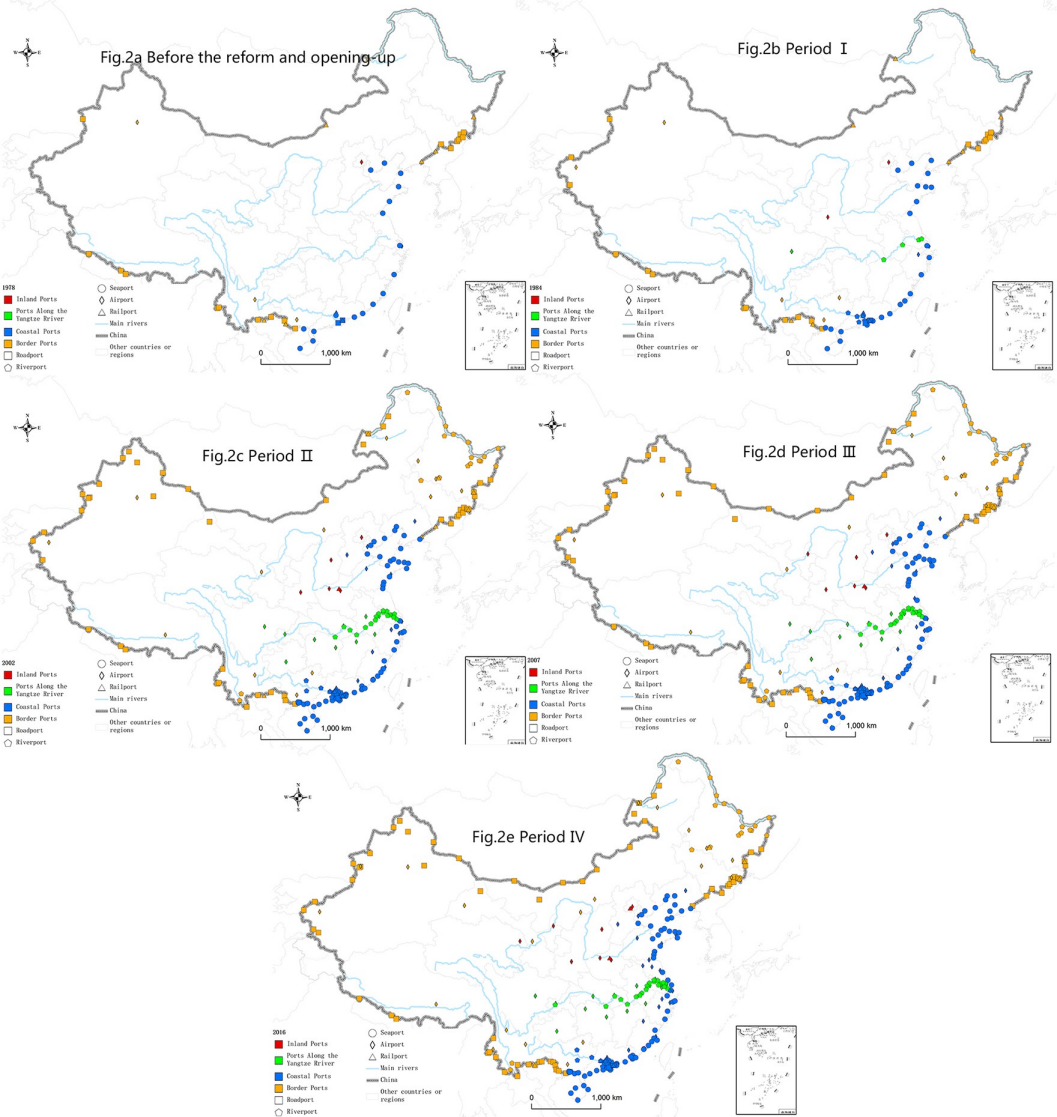
The changes in ports of entry distribution in the 40 years of reform and opening-up.

China has six maritime neighbors and has 14 land borders. Most of the ports of entry are located along the border and coastal areas, with a small portion located inland and along major rivers. Based on the spatial location, China's ports of entry can be divided into four open regional systems: Coastal Ports, Ports Along the Yangtze River, Border Ports and Inland Ports [15]. Period I, mainly coastal and river ports were opened; in Period II the number of border ports increased rapidly; in Period III, the overall growth rate slowed down; and in Period IV the number of ports in each region increased steadily (Fig 2).

The Coastal Ports include the ports of 11 provinces of Liaoning, Hebei, Tianjin, Shandong, Jiangsu, Shanghai, Zhejiang, Fujian, Guangdong, Guangxi and Hainan (except for riverports in Jiangsu, and border ports in Liaoning and Guangxi). The coastal areas have the most developed economies, the densest populations, and the highest openness degree in China. There are currently 140 ports in the coastal area, a six-fold increase over 1978. It had the fastest growth rate in Period I, far exceeding the national average growth rate over the same period. This is in line with the situation in which the coastal areas were first opened to the outside world. Seaports are the most important of all port types. At the beginning of the reform and opening-up period, the framework of the seaport system was already in place. After 40 years of development, there are now 81 seaports. A total of 15 riverports in the Coastal Ports are concentrated in the Pearl River basin of Guangdong and Guangxi. There are 29 airports in the coastal areas, which is 7 times more than that in 1978.

The Border Ports include the ports in the 9 border provinces of Liaoning, Jilin, Heilongjiang, Inner Mongolia, Gansu, Xinjiang, Tibet, Yunnan and Guangxi (except for the coastal ports in Guangxi and Liaoning). Most of the provinces along the border are far from the ocean. In the process of reform and opening-up, their development lagged the eastern coastal areas. Therefore, the opening process of the border ports has also lagged behind the coastal ports. In 1978, there were 27 border ports, slightly more than the number of coastal ports in the same period. Period II was the fastest growth period of the border ports, when 49 ports were added. Now, there are 116 border crossing ports, which is four times higher than in 1978. China has opened ports of entry with 12 of its 14 neighboring countries. Since China and Afghanistan have a short border and China has not yet established diplomatic relations with Bhutan, China has not opened a port with either country. For the provinces perspective, border ports are concentrated in Heilongjiang (25), Xinjiang (20), Inner Mongolia (18), Jilin (17) and Yunnan (18), accounting for 84% of the total. In terms of port types, roadports, railports and airports are widely distributed in various provinces, and riverports are concentrated in the Heilongjiang River Basin (16) and the Lancang River Basin (2).

The Ports Along the Yangtze River collectively includes 37 ports in the provinces along the Yangtze River (except the coastal ports). There are currently only two types of airports (15) and riverports (22). The Yangtze River Basin is one of the most important economic centers of China. After the reform and opening-up, a number of riverports and airports along the Yangtze River were quickly opened to the outside world. The riverports were mainly opened in Period I; the riverports and airports both had some growth in the latter three periods. Due to the lack of land ports and the relatively simple port types, the Ports Along the Yangtze River needs to be improved.

The Inland Ports include the ports in Beijing, Henan, Shanxi, Shaanxi, Ningxia and Qinghai. These areas do not border the sea, are not along big rivers, and do not border other countries, it is difficult to trade with foreign countries. So far, only the capitals and central cities of the provinces have opened airports, and Zhengzhou and Beijing have railports. With the implementation of the Belt and Road Initiative, these regions are expected to open new ports as part of the new international transportation corridor.

## Openness degree of ports of entry

### Results of POD

Kaiser Meyer Olkin (KMO) and Bartlett’s tests were used to assess the sampling adequacy before running PCA. When the sampling adequacy is greater than 0.5, the data set is suitable for running PCA [46, 56]. The original data were normalized using SPSS 24 software. The results of PCA are shown in Tables 2–4. In Table 2, the sampling adequacy is 0.625>0.50. Three components with eigenvalue larger than 1.00 were generated. The variable loadings larger than 0.30 or less than 0.30 are significant. In Table 4, all loadings with absolute values less than 0.8 were suppressed.

**Table 2:** Kaiser–Meyer–Olkin and Bartlett’s test.

Kaiser-Meyer-Olkin Measure of Sampling Adequacy.		0.625
Bartlett's Test of Sphericity	Approx. Chi-Square	1891.881
	df	21
	Sig.	0.000

**Table 3:** Total variance explained.

Component	Name	Eigenvalues	% of Variance	Cumulative %
1	FLI	3.014	43.053	43.053
2	IPI	2.091	29.868	72.921
3	CFA	1.054	15.052	87.973

**Table 4:** Rotated component matrix.

	FLI	IPI	CFA
PIL	0.876219		
PEL	0.864092		
EMC	0.817553		
PIP		0.988385	
POP		0.987616	
PCI			0.933766
PCE			0.90243

According to the component matrix obtained by PCA, multiplied by the data matrix obtained by the normalization to obtain a component score matrix. Since some of the scores have negative values, the matrix needs to be transformed. The transformed data order and eigenvalues are unchanged. The transformation formula is:

$$x\prime =\frac{x-x_{min}}{x_{max}-x_{min}}$$ (4)

In Eq 4, x' is the transformed new value, which is between 0 and 1; x is the original value, and x_max_ and x_min_ are the maximum and minimum values in the original data column, respectively. Taking the variance contribution of the rotated component as the weight, the POD score of each port is calculated (Table 5).

**Table 5:** Port openness degree.

Port	FLI	IPI	CFA	POD
Shenzhen Roadport	0.014851	0.523526	0.160768	0.699145
Shanghai Seaport	0.215162	0.008168	0.221868	0.445199
Zhuhai Roadport	0.002253	0.396437	0.022251	0.420941
Shanghai Airport	0.014579	0.102008	0.221868	0.338455
Shenzhen Seaport	0.11454	0.012822	0.160768	0.288131
Suzhou Riverport	0.082735	0.000519	0.146984	0.230238
Shanghai Railport	0.000432	0.000365	0.221868	0.222665
Beijing Airport	0.02156	0.071055	0.129905	0.22252
Tianjin Seaport	0.160859	0.002703	0.056431	0.219993
Qingdao Seaport	0.175959	0.001149	0.032348	0.209456
……				

Note: Only the top 10 of 207 ports are shown. See all POD scores and metadata in supporting information S1 Table.

### Comparative analysis between different ports

**Fig 3:**
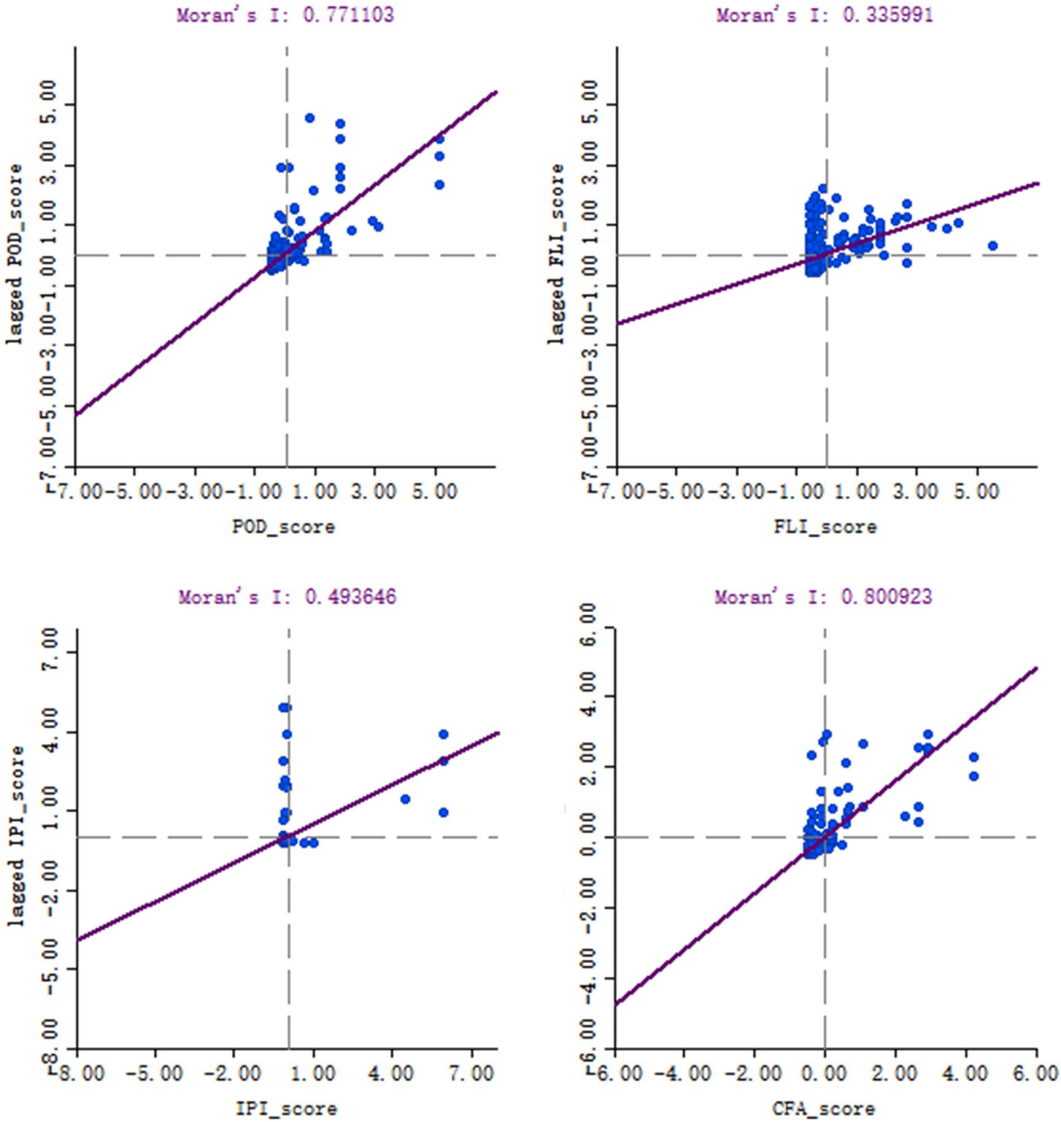
Moran’s I scatter plot of POD, FLI, IPI and CFA.

**Fig 4:**
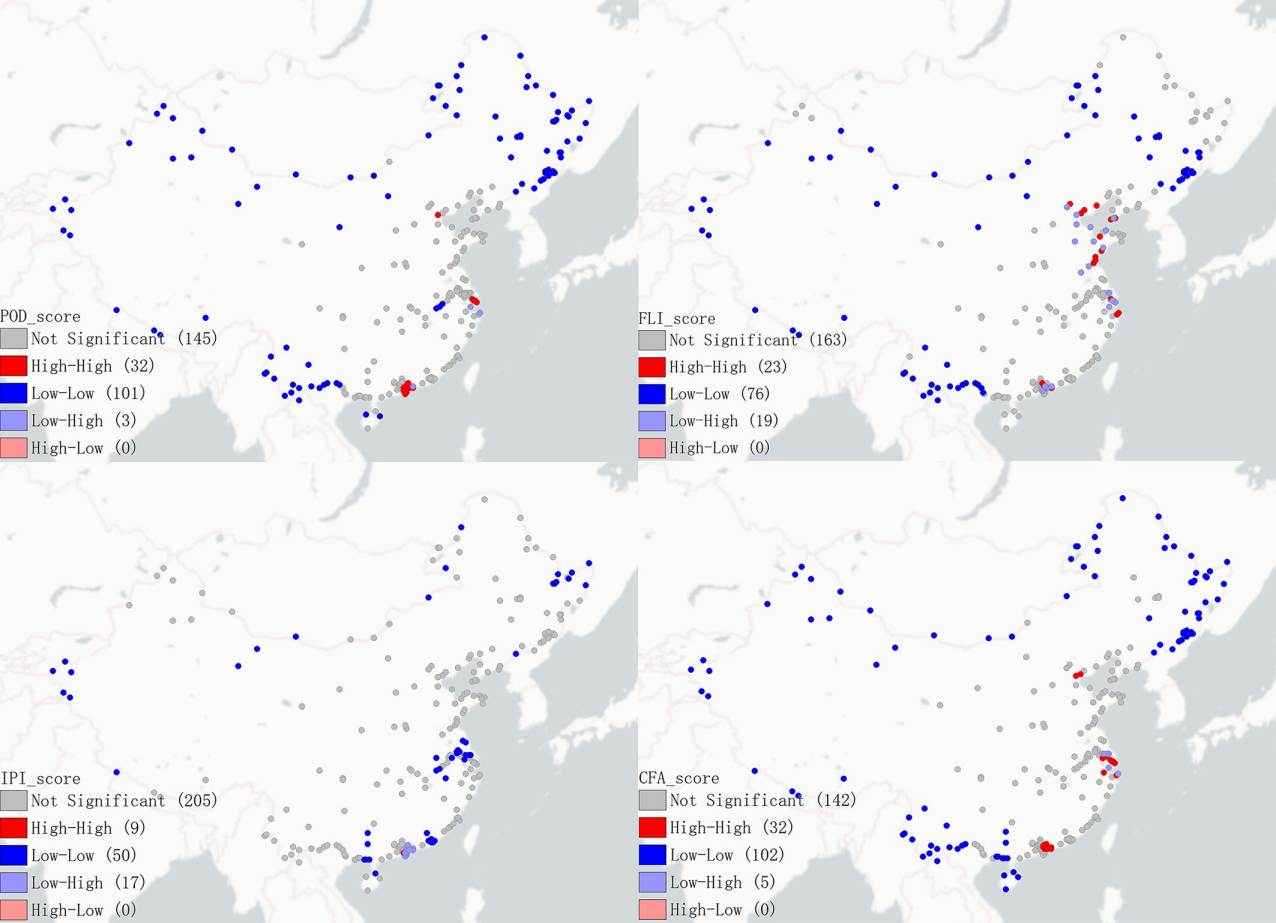
LISA scatter plot of POD, FLI, IPI and CFA.

Based on the results of POD, FLI, IPI and CFA, global Moran's I and local Moran's I were calculated, and the scatter plots of Moran's I and LISA (Figs 3 and 4) were drawn. The results of Tables 5 and 6 were analyzed with reference to Figs 3 and 4.

**Table 6:** POD average score of different types of ports.

		FLI	IPI	CFA	POD
Regional systems	Inland Ports	0.00491	0.00909	0.03139	0.04540
	Border Ports	0.00201	0.00098	0.00104	0.00403
	Coastal Ports	0.02559	0.01287	0.02746	0.06592
	Ports Along the Yangtze River	0.01063	0.00138	0.01462	0.02664
Port type	Roadport	0.00180	0.02830	0.00610	0.03621
	Seaport	0.04245	0.00114	0.01807	0.06167
	Airport	0.00338	0.00523	0.01716	0.02577
	Riverport	0.01477	0.00035	0.01251	0.02763
	Railport	0.00189	0.00081	0.03646	0.03917

#### FLI

The following characteristics can be seen from the scores of the port's FLI (Tables 5 and 6, Figs 3 and 4). ① The FLI generally has a positive spatial clustering, that is, ports with high (low) FLI values are concentrated together. High-High agglomeration is distributed in the Yangtze River Delta, Pearl River Delta and Bohai Rim region. Low-Low agglomeration is concentrated in the border areas. The agglomeration in other areas is not significant. ② The highest FLI in all of the ports is the Shanghai Seaport, which is 0.215 and is much higher than the other ports. ③ The FLI distribution is extremely uneven. There are 7 ports’ FLI are higher than 0.1, accounting for 3.38% of the sample and 41 in the range of 0.01–0.1, accounting for 19.81% of the sample. The trade volume of the first 7 ports accounted for 50.1% of the total, indicating that a small number of ports concentrated most of the country's foreign trade logistics. ④ The FLI of more than 76.8% ports is less than 0.01, that is, their foreign trade logistics flow is less than 1% of the Shanghai Seaport, indicating that the foreign trade flow of most ports is relatively small.

The results show the following: ① the FLI average values of the four port open regional systems are respectively: Coastal Ports (0.025) > Ports Along the Yangtze River (0.011) > Border Ports (0.005) > Inland Ports (0.002). The seaports concentrate most of the foreign trade logistics flow and are the main channel for China's foreign trade. Other types of port logistics volume are relatively low. ② Overall, the volume of import logistics is greater than the volume of export logistics. This is in line with the role of China as the world's factory. The northern ports have a higher logistics volume than the southern ports. This volume is related to the northern import and export of bulk cargo such as coal, iron and ore, which is in line with the industrial distribution of the south and the north. ③ The 4 seaports of Shanghai, Qingdao and Tangshan Rizhao constitute the first group, and their volume is above 270 million tons, far exceeding those of other ports. Except for the Weifang Seaports (6 tons), the foreign trade logistics volume of other shipping ports is more than 70,000 tons. The average volume of seaports is 56.48 million tons, with a median of 14.54 million tons. ④ The average foreign trade logistics volume of riverports is 10.18 million tons, with a median of 2.31 million tons. The 5 riverports of Suzhou, Wuxi, Nantong, Zhenjiang and Nanjing all have more than 20 million tons of logistics volume, all of which are in the Yangtze River Delta. Among them, the Suzhou Riverport has the largest logistics volume, reaching 135.23 million tons. Riverports with the largest logistics volume in the Pearl River Delta region are Foshan, Jiangmen, Zhongshan, and Dongguan. Their foreign trade logistics volume are all less than 7 million tons, and their overall scale is smaller than that in the Yangtze River Delta region. The Daxinganling Riverport has a volume of 16.44 million tons, which is the riverport with the highest volume in the Heilongjiang River Basin and the Lancangjiang River Basin. The volume of other riverports in these regions is less than 410,000 tons each. ⑤ In the airports, the volume of Shanghai, Beijing and Guangzhou far exceeds that of other airports. ⑥ Among the railports, the 4 major border-crossings of the China Railway Express train of Hulunbeier, Xilingol, Mudanjiang and Bortala have the largest volume. The Shenzhen Roadport and Yili Roadport are the largest roadports, with volume of more than 22 million tons, which is equivalent to the volume of medium-sized seaports.

#### IPI

The scores of the IPI are shown in Tables 5 and 6. The IPI mainly has the following characteristics: ① The IPI generally has a positive spatial clustering. High-High agglomeration is distributed in the Pearl River Delta region. Low-Low agglomeration is distributed in a small number of border areas and the middle and lower reaches of the Yangtze River. The agglomeration in other areas is not significant. ② The IPI average values of the four port open regional systems are respectively: Coastal Ports (0.013) > Inland Ports (0.009) > Ports Along the Yangtze River (0.0014) > Border Ports (0.001). ③ Shenzhen and Zhuhai, the 2 roadports connected to Hong Kong and Macao have the largest IPI, and the IPI is greater than 0.3. They are followed by the 3 airports of Shanghai, Beijing and Guangzhou. ④ The distribution of IPI is very uneven. Only 3 of them are larger than 0.1, and 196 of them are less than 0.01, indicating that most of the international passenger flow is concentrated in a few ports.

It can be seen from the IPI of different regions and types of ports that the overall international passenger flow is concentrated in the roadports and airports in the eastern coastal areas. The roadports on the China-Vietnamese and China-Myanmar borders, and the inland provincial capital city airports also have a large passenger flow.

#### CFA

The following characteristics can be seen from the scores of the port's CFA (Tables 5 and 6, Figs 3 and 4). ① The CFA generally has a positive spatial clustering. High-High agglomeration is distributed in the Yangtze River Delta, Pearl River Delta and Bohai Rim region. Low-Low agglomeration is concentrated in the border areas. The agglomeration in other areas is not significant. ② The CFA average values of the four port open regional systems are: Inland Ports (0.031) > Coastal Ports (0.027) >Ports Along the Yangtze River (0.014) > Border Ports (0.001). ③ The four port cities of Shanghai, Shenzhen, Suzhou and Beijing have the largest foreign trade volume. They are the central cities of China's 3 most developed urban agglomerations (the Yangtze River Delta, the Pearl River Delta and the Beijing-Tianjin-Hebei region). ④ The distribution of CFA is very uneven; it shows the scale of operations of port cities is quite different.

#### Comprehensive POD

Integrating the FLI, IPI and CFA, the POD (Tables 5 and 6, Figs 3 and 4) is obtained, which mainly draws the following conclusions. ① The POD generally has a positive spatial clustering. High-High agglomeration is distributed in the Yangtze River Delta, Pearl River Delta and Bohai Rim region. Low-Low agglomeration is concentrated in the border areas. The agglomeration in other areas is not significant. ② The POD average score of the four port open regional systems are: Coastal Ports (0.066) > Ports Along the Yangtze River (0.045) > Inland Ports (0.027) > Border Ports (0.004). ③ The POD of the port is a power law distribution. The highest POD of all ports is the Shanghai Seaport at 0.699. There are 21 ports with POD higher than 0.1, accounting for 10.1% of the sample. There are 112 ports with a POD of less than 0.01, accounting for 54% of the sample, which indicates that only a few ports have a high degree of openness. ④ The POD of a few ports is much higher than other ports, reflecting the hub role of these ports. ⑤ The high POD ports are mainly seaports with vast hinterlands and roadports connected to Hong Kong and Macao. The airports and riverports in the Pearl River Delta and Yangtze River Delta regions are mostly at medium levels. Most of the border ports and inland ports have low POD.

### Ports evolution tree

**Fig 5:**
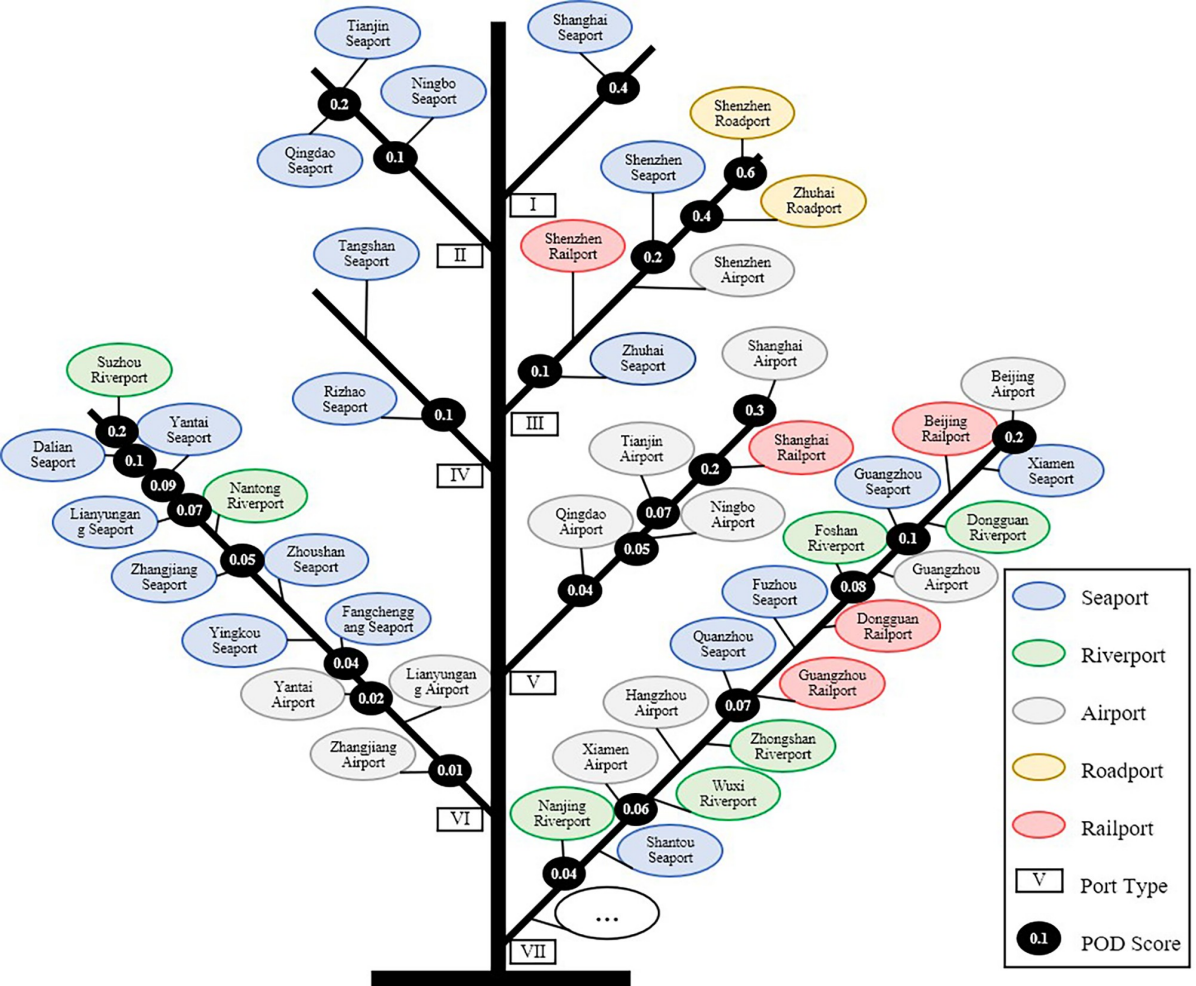
Ports evolution tree.

Using the K-means clustering algorithm, the ports are divided into 7 types. A total of 207 ports are arranged on the evolution tree, and each branch represents a type of ports. The ports on each branch are arranged according to the POD value. The ports far from the trunk have a high POD value, and the ports near the trunk have a low POD value (Fig 5). Shanghai seaport is the only Type I port, and it is a national hub port and the most open port in China. The Type II ports are regional hub ports, including the 3 seaports of Tianjin, Qingdao and Ningbo. The import and export passenger and cargo services of these ports are well developed, and the openness degree is second only to Shanghai seaport. The Type III ports are ports connecting Hong Kong and Macao, including six ports in the Pearl River Delta. They are the connecting channels between mainland China and Hong Kong and Macao. The Type IV ports are large ports for bulk minerals, and they include two seaports, Tangshan and Rizhao. They are China's main import and export ports for coal and various minerals. The Type V ports are ports that are close to the large hub ports, including the five ports of Shanghai Airport, Shanghai Railport, Tianjin Airport, Ningbo Airport and Qingdao Airport. These ports have similar geographical locations to Type I and Type II ports, and have higher openness degree. The Type VI ports are medium- and large-scale ports, including 12 ports. The passenger and cargo volume of such ports is also large, but it is significantly less than that of Type I and Type II ports. The Type VII ports are small ports. This type includes 178 ports. The passenger and freight traffic and the number of countries with which foreign trade is conducted by such ports is relatively small, so that the openness degree of these ports is relatively low.

### Impact factor analysis

We use geodetector to analyze the seven factors and the POD. Since the geodetector is suitable for the influence of the type variable on the dependent variable, seven factors are discretized in spss24.0 before calculation[52–54].

As shown in Table 7, the q value of each factor represents the degree to which the factor explains the spatial distribution of the POD. The PCE factor has the largest q value, followed by POP and PIP. PCE has a significant impact on the distribution of POD, indicating that the difference in export volume between different cities is large, and exports can significantly affect POD. The p value of PIL is the smallest, indicating that the difference in the amount of imported goods at the port has little effect on the spatial differentiation of POD.

**Table 7:** Results of factor q value.

	PEL	PIL	EMC	PCE	PCI	POP	PIP
q statistic	0.325198	0.196349	0.215447	0.529572	0.259194	0.418308	0.41748
p value	0.000	0.000	0.000	0.000	0.000	0.000	0.000

Geographical distribution characteristics are often the result of a combination of factors, so to explore the spatial differentiation of POD should consider multi-factor interaction. The interaction detector in geodetector can well explain the interaction of multi-factors with dependent variables. Among the 7 factors discussed in this study, the q values of the interaction of the two factors are all greater than the q value of the single factor. Among them, 18 pairs of factors enhance each other, and 3 pairs are nonlinear enhancement (Table 8). This shows that the spatial distribution difference of POD is determined by a combination of factors, and the explanatory power of a single factor is relatively weak.

**Table 8:** Results of interaction detector.

	PEL	PIL	EMC	PCE	PCI	POP	PIP
PEL	0.325198						
PIL	0.564157 ∽	0.196349					
EMC	0.687204 ∽	0.429265 ∽	0.215447				
PCE	0.691899 ↑	0.689406 ↑	0.74852 ↑	0.529572			
PCI	0.467601 ↑	0.455226 ↑	0.479662 ↑	0.559479 ↑	0.259194		
POP	0.666592 ↑	0.611794 ↑	0.602713 ↑	0.666905 ↑	0.58926 ↑	0.418308	
PIP	0.67235 ↑	0.614573 ↑	0.602858 ↑	0.674152 ↑	0.590077 ↑	0.434799 ↑	0.41748

Notes: ↑ denotes factors enhance each other; ∽ denotes nonlinear enhancement.

The results of ecological detector show that there are significant differences between some factors in terms of the effect on the spatial distribution of POD. As shown in Table 9, Y indicates a significant difference between the two factors, and N indicates that there is no significant difference. There is a significant difference between PCE and all other factors; there is a significant difference between POP and PIL, EMC, PCE and PCI; PIP also has significant differences with PIL, EMC, PCE and PCI.

**Table 9:** Results of ecological detector.

	PEL	PIL	EMC	PCE	PCI	POP
PIL	N					
EMC	N	N				
PCE	Y	Y	Y			
PCI	N	N	N	Y		
POP	N	Y	Y	Y	Y	
PIP	N	Y	Y	Y	Y	N

## Discussion and conclusion

Port development is an important part of China's reform and opening-up process. In the past 40 years, it has experienced four periods of startup, expansion, cooperation and optimization. In the startup period, the pilot open port policy provided a platform for foreign cooperation and exchange. During the expansion period, the port's opening-up process was expanded from the coastal ports to the inland ports, and the trade structure was continuously optimized. The experience gained in these ports helped China's accession to the World Trade Organization and its full participation in economic globalization. In the cooperation period, the port opening process was coordinated with China's overall industrial restructuring, which enhanced China's overall national strength, guaranteed the implementation of its WTO commitments, and improved the market economic system. During the optimization period, the port opening policy paid more attention to the balance, security and efficiency of opening up to the outside world, promoting the complementary advantages of inland and coastal areas, and providing guarantees for national strategies such as the Silk Road Economic Belt, the 21st Century Maritime Silk Road, and the Yangtze River Economic Belt. As gateways for China's opening-up to the outside world, the ports of entry followed the progress of the reform and opening-up, and each period had different development priorities. The port regional systems of the coast, the border, the river and the inland have been completed and the port system is still developing and improving.

The function of ports of entry is to connect internal and external markets, transport foreign trade goods, transport inbound and outbound passengers and facilitate foreign trade of the hinterland cities. Our measurement of the openness degree of ports of entry is also carried out in terms of a port’s foreign trade logistics intensity, international passenger intensity and foreign trade. The results show that the POD has significant type differences, geographical differences and quantitative differences. ① The POD generally has a positive spatial clustering. High-High agglomeration is distributed in the Yangtze River Delta, Pearl River Delta and Bohai Rim region. Low-Low agglomeration is concentrated in the border areas. The agglomeration in other areas is not significant. ② Among the four ports open regional systems, the POD of Coastal Ports is the highest followed by the Ports Along the Yangtze River, the Inland Ports, and the Border Ports, respectively. ③ Generally, the ports with high POD are concentrated in the eastern coastal areas and are mostly seaports. Low-POD ports are scattered in inland and border areas and are mostly roadports and railports. The polarization and difference in POD of different ports is obvious. The overall POD reflects a power law distribution. ④ The FLI of the seaports and riverports is the highest. The FLI in the Bohai Rim region is higher than those of the Yangtze River Delta region and the Pearl River Delta region. The overall difference in FLI is obvious, indicating that China's foreign trade logistics is concentrated in a few coastal ports. ⑤ The overall difference in IPI is obvious. The IPI of the roadports connected to Hong Kong and Macao in the Pearl River Delta region is the highest, followed by the airports of the 3 first-tier cities—Beijing, Shanghai, and Guangzhou—and then the airports of the inland provincial capital cities. ⑥ The largest port cities in terms of foreign trade are the core city of the Yangtze River Delta, the Pearl River Delta and the Beijing-Tianjin-Hebei region. ⑦ The analysis of the ports evolution tree shows that ports can be divided into 7 types. Type I ports are the national hub, Type II ports are the regional hub, Type III ports are the connecting Hong Kong and Macao, Type IV ports are the bulk mineral, Type V ports are ports close to the hub ports, Type VI ports are medium scale ports, and Type VII ports are the small ports. Type I to IV ports are large-scale ports with the highest openness degree. Type V ports also have high openness degree due to better geographical advantages; Type VI and Type VII ports have relatively small port sizes and low openness degree. ⑧ The results of geodetector analysis show that PCE can significantly affect the spatial differentiation of POD, while PIL has little effect on the spatial differentiation of POD. The spatial stratified heterogeneity of POD is determined by a combination of factors, and the explanatory power of a single factor is relatively weak. The results of ecological detector show that there are significant differences between some variables in terms of the effect on the spatial distribution of POD.

In summary, the eastern coastal ports have the highest openness degree, the openness of other types of ports is generally low, and the gap between them is patent. The seaports are close to the places of consumption and production, and international transportation is convenient, so the high-POD ports are mainly distributed in the coastal areas. Roadports and railports on the border usually only connect to a single country, and their openness is generally not high. Compared with the seaports, the roadports, railports, and airports have relatively low logistics volumes, and their POD are generally not high. The FLI, IPI and CFA are the responses to the broadness and intensity of the POD. Only a combination of these three can improve the comprehensive openness of the ports.

This paper analyzes the opening process of various types of ports from the perspective of time and space evolution, the interaction between the port development process and China's opening up policy from the perspective of time, and the distribution characteristics of ports and their relationship with cross-border passenger and cargo flows from a spatial perspective. In the context of economic globalization, international trade and cross-border logistics have developed rapidly, and the important role of ports has been highlighted. However, what followed was the unbalanced development of different regions and different types of ports, which hindered the coordinated development of the regional economies. This study starts from the spatial pattern of the ports and analyzes their degrees of openness in detail. It will help to solve the current dilemma of unbalanced port development. It can provide a theoretical basis for the balanced development of ports and the promotion of foreign economic and trade exchanges.

At the same time, this study may have the following shortcomings. We only considered the ports openness degree, and we did not fully describe the direction of its opening up, that is, which countries and regions are specifically connected. The study only describes the international passengers and logistics flow in general, and does not analyze the types of goods and passengers. The Belt and Road Initiative was proposed 5 years ago, and China’s opening-up situation has undergone new changes. Due to the lack of timeliness data, it has not been discussed in detail. These issues will continue to be explored in future studies.

## Supporting information

S1 Table Metadata and all POD scores. (XLSX)

S2 Table Details for the combination of similar ports. (XLSX)
